# Sorafenib-Induced Liver Failure: A Case Report and Review of the Literature

**DOI:** 10.1155/2011/941395

**Published:** 2011-12-12

**Authors:** Anneleen Van Hootegem, Chris Verslype, Werner Van Steenbergen

**Affiliations:** Liver Unit, Department of Pathophysiology, University Hospital Gasthuisberg, Catholic University of Leuven, 3000 Leuven, Belgium

## Abstract

In patients with hepatocellular carcinoma characterized by vascular invasion and/or extrahepatic disease, Sorafenib is considered treatment of choice. Although mild liver test abnormalities were reported in less than 1% of the patients in the two large randomized, controlled phase III trials, four cases of severe acute Sorafenib-induced hepatitis have been described. One of these four cases died from liver failure. In this paper, a patient with HCC with lung metastases developed high fever and a severe hepatitis that rapidly evolved into liver coma and death, two weeks after the initiation of Sorafenib. Biochemical parameters pointed to a hepatocellular type of injury. Clinical and biochemical presentations were compatible with a drug-induced hypersensitivity syndrome such as it has mainly been described for aromatic anticonvulsants, sulphonamides, and allopurinol. We hypothesize that an underlying cytochrome P450 dysfunction with the presence of reactive drug metabolites might lead to this potentially fatal Sorafenib-induced severe liver dysfunction.

## 1. Introduction

Hepatocellular carcinoma (HCC) is a highly prevalent malignancy with liver cirrhosis as the major predisposing factor. Choice of the treatment should be based on an individualised evaluation of each patient. It depends on multiple variables such as the size and number of tumoral nodules, degree of liver function, general health of the patient, the presence of portal invasion and of metastatic disease and requires a multidisciplinary approach [1–3]. In patients with advanced disease marked by vascular invasion and/or extrahepatic disease and presenting with an acceptable Child-Pugh score A or B and performance status not higher than 2, targeted therapy with the multikinase inhibitor Sorafenib is now considered the treatment of choice. This drug has been shown to lead to clinical relevant improvements in time to progression and in survival, with a magnitude of improvement in survival comparing with molecular-targeted therapies for other advanced cancers [1, 3, 4]. Sorafenib is considered to have an easily manageable associated toxicity without a treatment-related mortality [1, 4, 5].

As most patients who present with HCC have an underlying liver disease, an important issue related to this therapy is its potential liver toxicity. Liver dysfunction was reported in less than 1% of the Sorafenib-treated patients in the two randomized, double-blind, placebo-controlled multicentre, phase III trials (the SHARP trial and the Asia-Pacific trial) [6, 7]. Only four cases of Sorafenib-induced severe hepatitis have been described [8–11]. Liver toxicity occurred in two patients with underlying liver disease [8, 9] as well as in two cases with a previously normal liver function [10, 11]. One of the patients with a preexisting normal liver died of liver failure [10].

In this paper, we describe a patient with metastatic hepatocellular carcinoma who developed an acute fulminant hepatitis with fatal outcome two weeks after the start of treatment with Sorafenib. It is the purpose of the authors to bring this potential complication to the attention of hepatologists and oncologists involved in the treatment of HCC.

## 2. Case Report

A 76-year-old male Caucasian patient with a previous history of repeated episodes of acute alcoholic hepatitis was referred to our Liver Unit in June, 2007, because of the incidental finding on CT scan of a tumoral mass in segment IV of the liver. He was also known with arterial hypertension and with recurrent stomach ulcers for which he was treated since many years with perindopril and with omeprazole, respectively. On admission, the clinical examination was normal except for palmar erythema. Laboratory evaluation showed an alpha-foetoprotein (AFP) concentration of 525 *μ*g/L (nl < 14); haemoglobin was 14,7 g/dL, alkaline phosphatase 110 U/l (nl < 270), AST 46 U/l (nl < 38), ALT 39 U/l (nl < 41), and gamma-GT 54 U/l (nl < 53). Oesophageal varices were excluded, and a tumorectomy was performed. The resection specimen showed an undifferentiated hepatocellular carcinoma with vascular invasion; the nontumoral liver parenchyma was characterized by the presence of Mallory bodies and a septal stage of fibrosis. In October, 2008, and in February, 2009, radiofrequency ablation (RFA) was performed for recurrent HCC in segment IV. In February, 2009, previous to the second ablation therapy, AFP was 14 *μ*g/l, alkaline phosphatase 147 U/l, AST 63 U/l, ALT 45 U/l, and gamma-GT 104 U/l. There was no further use of alcohol since the diagnosis of cirrhosis, and HCC had been made.

In November, 2009, a repeated CT scan of thorax and liver was performed because of a rise in AFP up to 75 *μ*g/l; several small lung metastases were observed. In March, 2010, AFP had risen up to 743 *μ*g/l, and an increase in size of the lung metastases as well as a single, 17 mm large HCC-nodule in segment VII of the liver were visualized. At that time, alkaline phosphatase was 154 U/l, AST 125 U/l, ALT 60 U/l, and gamma-GT 304 U/l. Serum albumin was 46,9 g/dL, and the prothrombin time was 1,1 INR, indicative of a well-preserved liver function. The Child-Pugh status was A5. Sorafenib was started the first week of May, 2010, at a dosage of 400 mg per day during the first week and with an increase up to 800 mg daily from the second week of treatment onward. One week after the start of the 800 mg regime, the patient developed a flu-like syndrome with high spiking fever up to 40°C, tinnitus, nausea and severe vomiting, severe muscle cramps, anorexia, and watery diarrhea with a frequency of more than 10 stools per day. He was seen as an outpatient the first week of June, 2010. Clinical examination at that time revealed the presence of scleral jaundice and of a marked hepatomegaly, findings that had never been observed during previous examinations. Total bilirubin was 3,6 mg/dL with a direct fraction of 2 mg/dL; alkaline phosphatase was 135 U/l, AST 525 U/l, ALT 343 U/l, gamma-GT 215 U/l, ferritin 2099 *μ*g/l, albumin 3,2 g/l, and the prothrombin time was now 1,5 INR. The total white blood cell count was 7.4 × 10^9^/l with a neutrophil count of 5.7 × 10^9^/l; blood eosinophils were normal. Serology for hepatitis A, B, and C, as well as antinuclear antibodies were negative. Sonography showed a large liver without focal liver lesions or dilated bile ducts and with only a minimal amount of ascites so that a diagnostic paracentesis was not considered to be useful. As the diarrhea had already improved at the time of the consultation, stool cultures have not been performed. Sorafenib was stopped but the patient went rapidly into a severe jaundice and a deep liver coma and died on June 16, 2010. As his family refused any further hospitalisation, we were not able to perform a liver biopsy.

## 3. Discussion

In patients with advanced hepatocellular carcinoma characterized by vascular invasion and/or extrahepatic disease and presenting with an acceptable Child-Pugh score A or B, Sorafenib is considered the treatment of choice. Sorafenib is an oral multikinase inhibitor that blocks tumor cell proliferation by targeting Raf/MEK/ERK signalling pathway and that has an antiangiogenic effect by targeting vascular endothelial growth factor receptor [5, 6]. Overall, oral Sorafenib is a well-tolerated treatment option with an acceptable safety profile with hand-foot skin reaction and diarrhea as the most frequent side effects [6, 7]. Liver dysfunction was reported in less than 1% of the Sorafenib-treated patients in the SHARP trial and the Asia-Pacific trial [6, 7]. Outside the mild liver dysfunction reported in both these randomized, placebo-controlled multicentre, phase III trials, four cases of Sorafenib-induced severe hepatitis have been described [8–11]. Liver toxicity occurred in two patients with underlying liver disease [8, 9] as well as in two cases with a previously normal liver function, already suggesting an idiosyncratic drug reaction [10, 11]. One of the patients with a preexisting normal liver died of liver failure [10]. Here, we report a second patient with an apparent Sorafenib-induced liver failure resulting in death shortly after the start of Sorafenib therapy. 

Our patient presented with a clinical-biochemical picture of an acute hepatitis with severe general symptoms consisting of high fever, anorexia and vomiting, watery diarrea, and severe muscle cramps, and with a marked rise in aminotransferases about ten times above the upper limit of normal. These symptoms occurred two weeks after the start of Sorafenib and one week after increasing the dosage from 400 to 800 mg daily. In three other cases reported, Sorafenib-induced hepatitis also presented with flu-like symptoms with fever [8] or high fever up to 39°C [11], with skin rash and diarrhea [8], nausea [11], with a hepatocellular type of injury with high transaminases [9, 11], and with a histological picture of hepatocellular centrolobular necrosis [8, 11]. In our patient, the hepatocellular type of injury was also evident from a high R ratio of 16,8, as calculated from the ratio (ALT/ULN)/(alkaline phosphatase/ULN), with a ratio >5 indicating hepatocellular injury and <2 cholestatic injury [12]. Besides the symptoms such as fever and rash and the early onset within one to eight weeks after Sorafenib-introduction reported in our and in other cases, the presence of eosinophilic infiltration in the portal tracts and a positive lymphocyte transformation test that was subsequently performed in the patient reported by Herden et al. [11] suggests an allergic, drug-induced hypersensitivity syndrome (DHIS) as the pathogenetic mechanism of the Sorafenib-induced hepatitis [12–16]. Interestingly, in the patient with severe Sorafenib-induced hepatitis reported by Schramm et al. [8], prednisolone was given as a treatment for the acute hepatitis and transaminases returned to baseline levels within 10 days. The management of DHIS involves discontinuation of the offending drug as well as the administration of moderate to high doses of systemic glucocorticosteroids, especially in cases with extensive involvement of the internal organs such as myocarditis [13, 14]. In our case, Sorafenib was stopped but the patient rapidly went into a deep liver coma and the family refused any further hospitalisation, so that steroid treatment could not be given. Because of this refusal for hospitalisation, it was also not possible to perform a liver biopsy. In our opinion, however, the clinical evolution as well as the biochemical findings occurring shortly after the start of Sorafenib treatment were sufficient to make a diagnosis of a severe drug-induced liver damage obvious.

A severe drug reaction with systemic symptoms can also be part of the so-called DRESS syndrome or drug reaction with eosinophilia and systemic symptoms; this syndrome is not always characterized by eosinophilia, and its main features are skin rash with a diversity of cutaneous eruptions, high fever, and organ involvement with, amongst others, hepatomegaly and hepatitis with potential evolution towards liver failure and death. Other noteworthy features are a delayed onset, usually 2–6 weeks after initiation of the drug, and the possible persistence or aggravation of symptoms despite discontinuation of the culprit drug [15, 16]. Although our patient did not show any skin eruption, the high fever with hepatomegaly and death from liver failure might be compatible with DRESS syndrome. Moreover, one patient that was previously reported with Sorafenib hepatitis did show a cutaneous rash [8], whereas an eosinophilic infiltration in portal tracts was found in another case [11]. Also for the DRESS syndrome with internal organ involvement, treatment with systemic corticosteroids is proposed [15, 16].

The drug-induced hypersensitivity syndrome and the DRESS syndrome are most frequently related to the use of antiepileptic agents such as carbamazepine, phenobarbital, and phenytoin; antibiotics such as sulphonamides; dapsone, sulfasalazine, and allopurinol [14, 15]. Biologically reactive metabolites are thought to be responsible for a delayed immunologically mediated reaction with macrophage and T-lymphocyte activation and cytokine release [13, 14]. Defects in detoxification enzymes for aromatic anticonvulsants, slow acetylator phenotype, and an increased susceptibility of lymphocytes to toxic metabolites in the case of sulphonamides have been implicated in the pathogenesis of these clinical conditions [15, 16]. The oxidative metabolism of Sorafenib occurs primarily in the liver and is mediated by the cytochrome P450 (CYP)3A4. Eight Sorafenib metabolites have been identified with pyridine N-oxide being the main circulating metabolite in plasma [5]. It could be hypothesized that an underlying cytochrome P450 dysfunction and the presence of biologically reactive drug metabolites might lead to this Sorafenib-induced severe liver dysfunction. DHIS and DRESS have also been associated with human herpesvirus 6 (HHV-6) reactivation [17, 18], and, according to Shiohara et al. [17], HHV-6 reactivation is even one of their 6 diagnostic criteria for DIHS/DRESS. In this regard, the clinical symptoms with high fever observed during the course of DIHS/DRESS could be mediated by antiviral T cells, which could explain amongst others a further deterioration of liver function after withdrawal of the offending drug [17]. HHV-6 serology and PCR-DNA have therefore been applied in the diagnosis of patients with these conditions [17, 18]. Serologic examination for HHV-6 has not been performed in our patient.

In conclusion, it could be suggested to add Sorafenib to the list of drugs that may lead to the drug-induced hypersensitivity syndrome and possibly also to the DRESS syndrome. The appearance of high fever and liver test abnormalities after initiation of Sorafenib should lead to an immediate discontinuation of the drug and to the initiation of therapy with corticosteroids in case of severe hepatitis. In case of detection of further cases of severe Sorafenib-induced hepatitis in patients with HCC, a study of HHV-6 antibodies and DNA could be useful to study the possible role of HHV-6 reactivation in the pathogenesis of this potentially lethal liver disease.
